# Contrast-enhanced carotid ultrasound improves vulnerable plaque detection in acute central retinal artery occlusion: a propensity score–matched study

**DOI:** 10.3389/fcvm.2025.1664769

**Published:** 2026-01-05

**Authors:** Zhen Song, Changjiang Zhou, Na Yang, Zhenyi Huang, Bin Li, Yunpeng Si

**Affiliations:** 1 Department of Ultrasound, Jinan people’s Hospital Affiliated to Shandong First Medical University, Jinan, Shandong, China; 2 Department of Ophthalmology, Jinan people’s Hospital Affiliated to Shandong First Medical University, Jinan, Shandong, China

**Keywords:** central retinal artery occlusion, contrast-enhanced ultrasound, carotid plaques, embolic risk, propensity score matching

## Abstract

**Background:**

Central retinal artery occlusion (CRAO) is an ophthalmic emergency that signals a markedly increased risk of ischemic stroke and systemic vascular events. Contrast-enhanced ultrasound (CEUS) offers advanced imaging of carotid plaque vulnerability, but its diagnostic utility in CRAO remains inadequately explored.

**Objective:**

To evaluate the diagnostic value of CEUS in identifying vulnerable carotid plaques in patients with acute CRAO.

**Methods:**

In this retrospective, propensity score–matched study, 110 patients with acute CRAO and 110 matched healthy controls were enrolled. Matching was performed for age, sex, and key cardiovascular risk factors. All participants underwent standardized carotid duplex ultrasound and CEUS, with plaque vulnerability assessed by intraplaque neovascularization grading. Logistic regression was used to identify independent predictors of CRAO. Diagnostic performance was evaluated using receiver operating characteristic (ROC) curve analysis.

**Results:**

Compared to controls, CRAO patients had a significantly higher prevalence of vulnerable carotid plaques (CEUS enhancement scores ≥2: 85.5% vs. 21.8%, *p* < 0.001). Key ultrasound predictors of CRAO included increased intima-media thickness, plaque length, higher peak systolic velocity, resistance index, pulsatility index, and CEUS enhancement score. The combined diagnostic model, integrating both conventional and CEUS parameters, demonstrated superior accuracy (AUC = 0.916) compared to conventional ultrasound (AUC = 0.738) or CEUS alone (AUC = 0.754).

**Conclusions:**

CEUS significantly improves the detection of vulnerable carotid plaques in patients with acute CRAO and, when combined with conventional ultrasound, markedly enhances diagnostic performance. Incorporating CEUS into routine vascular assessment may facilitate better risk stratification and inform personalized secondary prevention strategies for CRAO patients.

## 1 Introduction

Central retinal artery occlusion (CRAO) is an ophthalmic emergency characterized by sudden, painless monocular vision loss due to obstruction of the central retinal artery (1, 2). While traditionally considered an isolated ocular event, CRAO is now increasingly recognized as an indicator of elevated risk for subsequent ischemic stroke and cardiovascular disease (3). Accordingly, current guidelines classify CRAO as a subtype of ocular stroke, necessitating urgent vascular assessment and the implementation of secondary prevention strategies (4). Embolic events account for a significant proportion of CRAO cases, with unstable atherosclerotic plaques in the ipsilateral carotid artery frequently serving as the embolic source (5). While conventional duplex ultrasonography can assess the degree of luminal stenosis, it provides limited information regarding plaque composition and vulnerability—key factors in determining embolic potential (6).

Contrast-enhanced ultrasound (CEUS) addresses these limitations by delineating plaque morphology and visualizing intraplaque neovascularization—an established marker of plaque instability (7, 8). Although CEUS is increasingly used for risk stratification in ischemic stroke and cardiovascular disease, its clinical utility in CRAO remains insufficiently characterized (9).

In this propensity score–matched case-control study, we aimed to evaluate the diagnostic utility of CEUS for identifying vulnerable carotid plaques in patients with acute CRAO, as compared to matched healthy controls.

## 2 Methods

### 2.1 Study design and participants

**Figure 1:**
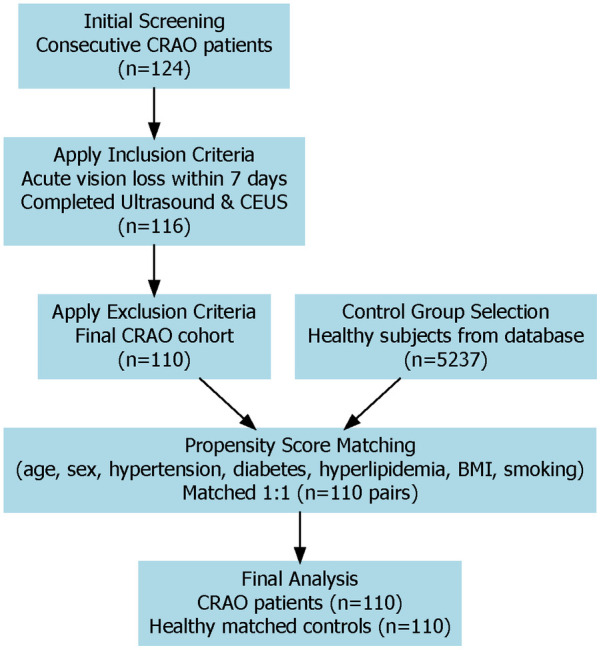
Study flow chart of patient selection and matching. This diagram shows how patients with CRAO and healthy controls were chosen for the study. After applying inclusion and exclusion criteria, 110 CRAO patients and 110 matched controls were enrolled using propensity score matching. Both groups received carotid ultrasound and CEUS examinations.

We conducted a retrospective observational study at a tertiary medical institution. All consecutive patients diagnosed with acute CRAO between January, 2023, and December, 2024, were systematically screened for eligibility. The diagnosis of CRAO was established through comprehensive clinical assessments and fundus fluorescein angiography in adherence to the 2021 American Heart Association Scientific Statement guidelines. Following stringent inclusion and exclusion criteria, 110 patients with confirmed CRAO constituted the case cohort. The control group consisted of 110 healthy individuals selected from the hospital's health examination database, matched 1:1 with CRAO patients by age, sex, and major cardiovascular risk factors via propensity score matching, ensuring balanced baseline characteristics. The study protocol was reviewed and approved by the Institutional Review Board of Jinan People's Hospital (Approval No. 2025-LW-045). See Figure 1 for the detailed flow chart.

### 2.2 Inclusion and exclusion criteria

Eligible patients presented with acute-onset monocular vision loss within seven days, had CRAO confirmed by ophthalmologic evaluation and angiographic findings, and underwent bilateral carotid duplex ultrasound and CEUS within 72 h of diagnosis. Exclusion criteria encompassed prior carotid interventions (endarterectomy or stenting), known autoimmune vasculitis or coagulopathies, cardiac embolic sources such as atrial fibrillation with documented left atrial thrombus, or incomplete clinical or ultrasound data.

### 2.3 Propensity score matching methodology

To mitigate selection bias and ensure comparability of baseline characteristics, propensity scores were computed using logistic regression models. Included covariates were age, sex, hypertension, diabetes mellitus, hyperlipidemia, body mass index, and current smoking status. We utilized nearest-neighbor matching with a caliper width of 0.2 standard deviations and no replacement, ultimately achieving 110 balanced pairs for analysis.

### 2.4 Carotid duplex ultrasound and CEUS protocol

All participants underwent standardized carotid duplex ultrasound using a 7–15 MHz linear array transducer. The following parameters were systematically evaluated: intima-media thickness (IMT), plaque presence and morphology, plaque length according to NASCET criteria, peak systolic velocity (PSV), resistance index (RI), and pulsatility index (PI).

Immediately after conventional ultrasound assessment, CEUS was performed using an intravenous bolus of 2.4 ml SonoVue contrast agent, maintaining a low mechanical index (<0.10) during three minutes of continuous imaging. Intraplaque neovascularization was evaluated via a validated semi-quantitative grading system: grade 0 (no enhancement), grade 1 (punctate enhancement), grade 2 (punctate enhancement with one to two short linear foci), and grade 3 (linear or extensive traversing enhancement). Plaques receiving grades 2 or 3 were categorized as “vulnerable,” indicative of substantial intraplaque neovascularization and heightened embolic potential, in accordance with previously validated criteria (10). Two experienced, board-certified sonographers independently performed plaque grading, blinded to clinical data, with adjudication by a third senior reviewer in cases of discrepancy to ensure high interobserver reliability.

### 2.5 Sample size estimation

Based on preliminary data suggesting a 40% difference in vulnerable plaque prevalence between CRAO patients and controls, a minimum of 94 subjects per group was required for 90% power at a two-sided alpha of 0.05. To account for potential attrition, 110 participants were enrolled in each group.

### 2.6 Statistical methods

Statistical analyses were conducted using R software (version 4.3.1). Continuous variables were expressed as mean ± standard deviation (SD) or median (interquartile range, IQR) and compared using *t*-tests or Mann–Whitney *U* tests as appropriate. Categorical variables were presented as frequencies and percentages, analyzed by chi-square or Fisher's exact test. Binary logistic regression identified independent predictors of CRAO, reported as odds ratios (OR) with 95% confidence intervals (CI). Diagnostic performance was evaluated using receiver operating characteristic (ROC) curves and area under the curve (AUC). A two-sided *p*-value <0.05 was considered statistically significant.

## 3 Results

### 3.1 Baseline clinical characteristics

A total of 220 subjects were included, comprising 110 patients with acute CRAO and 110 healthy controls matched for age, sex, and major cardiovascular risk factors using propensity score matching (Table 1). The two groups were well balanced in terms of demographic features and baseline cardiovascular risk profiles, with no significant differences in age, sex distribution, BMI, hypertension, diabetes, smoking, or alcohol use (all *p* > 0.05). Laboratory parameters—including white blood cell, neutrophil, lymphocyte, platelet, fibrinogen, and HDL cholesterol—were also comparable. Notably, the CRAO group exhibited higher levels of creatinine, uric acid, triglycerides, total cholesterol, and LDL cholesterol (all *p* < 0.05); however, most standardized mean differences remained below 0.2, indicating minimal residual imbalance. Overall, the two groups were sufficiently comparable, minimizing potential confounders before carotid ultrasound evaluation.

**Table 1:** Baseline characteristics.

Variable	Level	Control (*N* = 110)	CRAO (*N* = 110)	*t/x* ^2^	*p*-value	SMD
Gender (%)	Female	43 (39.1)	45 (40.9)	0.019	0.891	0.037
	Male	67 (60.9)	65 (59.1)			
Age [mean (SD)]		56.89 (9.49)	56.46 (11.84)	0.301	0.768	0.040
BMI [mean (SD)]		26.02 (3.20)	26.30 (3.59)	−0.611	0.541	0.082
Hypertension (%)	No	57 (51.8)	61 (55.5)	0.165	0.685	0.073
	Yes	53 (48.2)	49 (44.5)			
Diabetes (%)	No	95 (86.4)	92 (83.6)	0.143	0.706	0.076
	Yes	15 (13.6)	18 (16.4)			
Smoking (%)	No	94 (85.5)	97 (88.2)	0.159	0.690	0.081
	Yes	16 (14.5)	13 (11.8)			
Drinking (%)	No	101 (91.8)	101 (91.8)	0.000	1.000	<0.001
	Yes	9 (8.2)	9 (8.2)			
WBC [mean (SD)]		6.32 (1.67)	6.09 (1.75)	1.00	0.326	0.133
Neutrophil [mean (SD)]		3.55 (1.19)	3.64 (1.29)	−0.54	0.596	0.072
Lymphocyte [mean (SD)]		1.78 (0.56)	1.83 (0.64)	−0.62	0.536	0.084
Platelet [mean (SD)]		210.97 (49.17)	201.66 (59.34)	1.27	0.207	0.171
Creatinine [mean (SD)]		63.23 (12.70)	73.96 (22.41)	−4.37	<0.001	0.589
Triglyceride [mean (SD)]		1.43 (0.77)	1.87 (0.93)	−3.82	<0.001	0.517
Total Cholesterol [mean (SD)]		4.50 (0.91)	4.77 (1.03)	−2.06	0.045	0.271
HDL [mean (SD)]		1.12 (0.30)	1.09 (0.27)	0.78	0.413	0.111
LDL [mean (SD)]		2.59 (0.66)	2.84 (0.95)	−2.27	0.030	0.294
Fibrinogen [mean (SD)]		2.88 (0.62)	2.96 (0.67)	−0.92	0.393	0.115

### 3.2 Carotid ultrasound and CEUS findings

Building on the baseline comparability of the study cohorts, conventional carotid duplex ultrasound revealed that CRAO patients had significantly increased IMT, longer plaque length, and higher PSV, RI, and PI compared to controls (all *p* < 0.001). The prevalence of carotid plaque was also markedly higher among CRAO patients (84.5% vs. 40.0%).

To further characterize plaque vulnerability, CEUS was performed in both groups. A significantly higher proportion of CRAO patients (85.5%) demonstrated plaques with CEUS enhancement scores ≥2, compared to 21.8% in the control group (*p* < 0.001), see Table 2.

**Table 2:** Comparison of carotid ultrasound parameters between control and CRAO groups.

Variable	Level	Control	CRAO	*t/x* ^2^	*p*-value
IMT [mean (SD)]		0.84 (0.15)	1.11 (0.18)	−12.09	<0.001
Plaque (%)	No	66 (60.0)	17 (15.5)	44.577	<0.001
	Yes	44 (40.0)	93 (84.5)		
Plaque_Length [mean (SD)]		7.40 (1.68)	11.36 (2.90)	−12.39	<0.001
PSV [mean (SD)]		96.50 (15.29)	124.84 (20.11)	−11.77	<0.001
RI [mean (SD)]		0.62 ± 0.08	0.72 ± 0.09	−8.75	<0.001
PI [mean (SD)]		0.99 (0.18)	1.40 (0.29)	−12.60	<0.001
CEUS_Enhancement_Score (%)	0	45 (40.9)	5 (4.5)	93.432	<0.001
	1	41 (37.3)	11 (10.0)		
	2	20 (18.2)	58 (52.7)		
	3	4 (3.6)	36 (32.7)		
Neovascularization_HighRisk (%)	Low	86 (78.2)	16 (14.5)	87.024	<0.001
	High	24 (21.8)	94 (85.5)		

### 3.3 Independent predictors of CRAO

**Figure 2:**
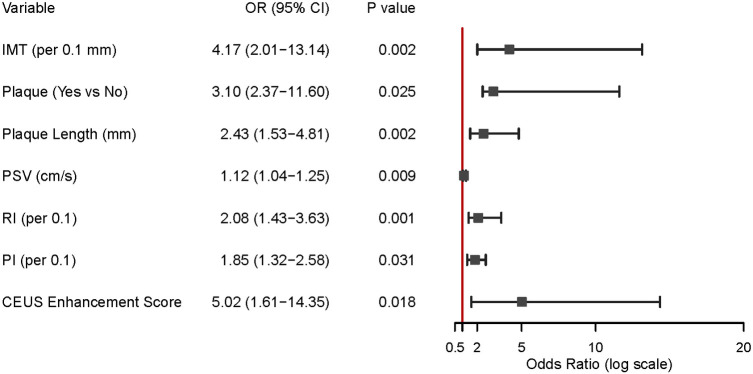
Key carotid ultrasound predictors of CRAO. This figure displays the results of logistic regression analysis, identifying factors that increase CRAO risk. Important predictors include thicker intima-media, the presence and length of carotid plaque, higher peak systolic velocity (PSV), resistance index (RI), pulsatility index (PI), and higher CEUS enhancement scores. All of these measures were independently linked with a greater risk of CRAO.

To further delineate the independent risk factors for CRAO, we performed a binary logistic regression analysis incorporating key carotid ultrasound and CEUS parameters as listed in Figure 2. The results demonstrated that IMT was independently associated with CRAO, with each 0.1 mm increase in IMT conferring a more than fourfold increase in risk (OR = 4.17, 95% CI: 2.01–13.04, *p* = 0.002). The presence of carotid plaque (OR = 3.10, 95% CI: 2.37–11.60, *p* = 0.025), greater plaque length (OR = 2.43 per mm, 95% CI: 1.53–4.81, *p* = 0.002), and higher peak systolic velocity (PSV) (OR = 1.12 per cm/s, 95% CI: 1.04–1.25, *p* = 0.009) were also significant independent predictors of CRAO.

Additionally, both RI (OR = 2.08 per 0.1 increase, 95% CI: 1.43–3.63, *p* = 0.001) and PI (OR = 1.85 per 0.1 increase, 95% CI: 1.32–2.58, *p* = 0.031) were associated with increased CRAO risk. Notably, a higher CEUS enhancement score was a strong independent risk factor for CRAO (OR = 5.02, 95% CI: 1.61–26.35, *p* = 0.018). (See Figure 2).

### 3.4 ROC analysis for diagnostic value

ROC curve analysis was conducted to assess the diagnostic value of different carotid ultrasound models for CRAO (see Table 2 for parameter definitions). The conventional ultrasound model, which included IMT, plaque presence, plaque length, PSV, RI, and PI, demonstrated moderate discriminatory power with an AUC of 0.738. The CEUS-only model, incorporating the CEUS enhancement score as an indicator of intraplaque neovascularization, provided a slightly higher AUC of 0.754, reflecting improved sensitivity and specificity compared to conventional ultrasound alone.

**Figure 3:**
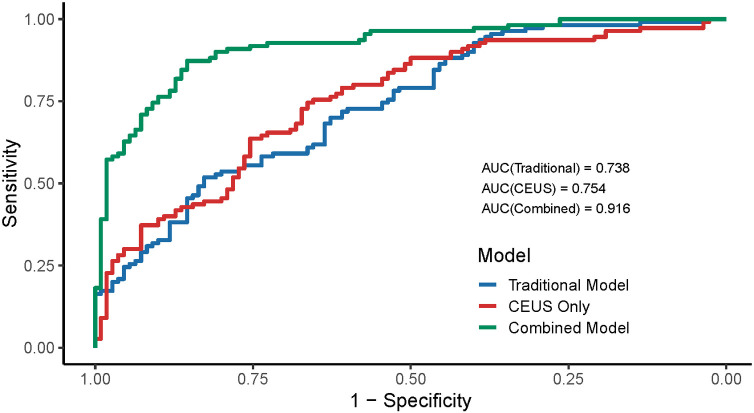
Diagnostic accuracy of ultrasound and CEUS for CRAO. This graph compares the accuracy of three models for diagnosing CRAO: conventional ultrasound alone, CEUS alone, and both methods combined. The combined model had the highest area under the curve (AUC = 0.916), meaning it best distinguishes CRAO patients from controls. Adding CEUS to standard ultrasound greatly improves diagnostic performance.

Crucially, the combined diagnostic model, which integrated both conventional ultrasound parameters and CEUS-derived measures, achieved the highest diagnostic accuracy, with an AUC of 0.916 (see Figure 3). These results demonstrate that while both conventional and contrast-enhanced ultrasound independently aid in non-invasive CRAO detection, integrating CEUS significantly improves diagnostic performance.

## 4 Discussion

In this propensity score–matched observational study, we demonstrated that CEUS significantly enhances the detection of vulnerable carotid plaques in patients presenting with acute CRAO. Notably, CRAO patients exhibited a substantially higher prevalence of plaques graded ≥2 for intraplaque neovascularization compared to healthy controls (85.5% vs. 21.8%). Furthermore, integrating CEUS-derived parameters with conventional ultrasound measures markedly improved diagnostic accuracy, achieving an AUC of 0.916. These findings robustly support our hypothesis that CEUS provides incremental value in identifying high-risk plaques and refining embolic risk stratification in CRAO (11).

Our results extend and reinforce existing literature underscoring CEUS's role in vascular risk stratification, particularly within cerebrovascular pathology. Although widely implemented in assessing symptomatic carotid plaques linked to ischemic stroke, CEUS's application in CRAO remains comparatively under-investigated. Previous studies have documented that CEUS effectively identifies intraplaque neovascularization in 60%–70% of symptomatic plaques, even absent significant stenosis (12). Aligning closely with data derived from acute ischemic stroke populations, our study confirms that elevated CEUS enhancement scores independently correlate with CRAO. This observation highlights the clinical significance of plaque microangiogenesis as an indicator of embolic potential specifically within ophthalmic circulation. The enhanced diagnostic precision achieved through CEUS, particularly when complemented by conventional imaging techniques, aligns with contemporary multicenter findings in stroke research (13).

Moreover, our findings indicate that CRAO patients exhibit significantly elevated serum creatinine, triglycerides, and LDL cholesterol levels relative to healthy controls. Elevated creatinine levels may reflect underlying renal dysfunction, a condition intrinsically linked to heightened systemic vascular risk due to accelerated atherosclerotic progression and increased vascular inflammation (14). Similarly, elevated triglycerides likely contribute to plaque instability by promoting lipid accumulation and inflammatory processes within arterial walls (15). Increased LDL cholesterol levels further underscore a well-established relationship with accelerated atherosclerotic plaque formation, serving as a critical, modifiable risk factor for cardiovascular morbidity (16). These biochemical differences further support the concept of CRAO as a systemic vascular event with underlying metabolic dysregulation, rather than an isolated ocular condition.

The underlying pathophysiology supporting these findings is biologically coherent. Vulnerable carotid plaques frequently exhibit extensive immature microvascular networks arising from the vasa vasorum, predisposing plaques to hemorrhage, lipid-rich core expansion, and potential fibrous cap disruption (17). CEUS uniquely facilitates the dynamic visualization of these neovessels, providing nuanced insights into plaque instability beyond traditional structural imaging modalities (18). Additionally, observed elevations in intima-media thickness, plaque length, peak systolic velocity, resistance index, and pulsatility index in CRAO patients indicate significant underlying structural and hemodynamic disturbances conducive to microembolization. These cumulative findings reinforce the conceptualization of CRAO as a manifestation of systemic vascular disease rather than a purely ocular event.

Clinically, our findings advocate for the incorporation of CEUS as a minimally invasive imaging tool to enhance early risk stratification for recurrent embolic episodes in CRAO. By identifying high-risk plaques that may evade detection via conventional duplex ultrasound—typically reliant solely on luminal stenosis assessment—CEUS could facilitate individualized secondary prevention strategies (19). Specifically, extensive intraplaque neovascularization detection could prompt intensified antiplatelet or lipid-lowering regimens and, selectively, consideration for carotid revascularization even in the absence of significant stenosis (20). Consequently, CEUS emerges as a valuable imaging biomarker reflective of systemic atherosclerotic burden, holding potential to substantially mitigate subsequent cerebrovascular and cardiovascular events in this vulnerable patient population (21, 22). From a healthcare economics perspective, while CEUS adds incremental cost to routine carotid assessment, the potential to prevent recurrent strokes—which carry substantial healthcare costs and disability burden—may justify this investment. Formal cost-effectiveness analyses incorporating stroke prevention rates, quality-adjusted life years, and healthcare utilization costs would be valuable to inform healthcare policy decisions regarding CEUS implementation in routine CRAO evaluation (23, 24).

Despite these compelling results, our study has several limitations that should be considered. First, as a single-center retrospective study, the generalizability of our findings is limited and subject to inherent biases. However, we attempted to mitigate these issues through rigorous propensity score matching and standardized imaging protocols to enhance internal validity. Second, the interpretation of CEUS requires specialized expertise, which may hinder widespread clinical adoption. Nevertheless, the high interobserver reliability observed in this study suggests that consistent results can be achieved with proper training and experience. Finally, our analysis focused solely on imaging-based phenotypes and did not include long-term clinical outcomes. Therefore, prospective, multicenter studies are needed to validate our findings, determine the prognostic value of CEUS-derived neovascularization scores in CRAO, and assess the long-term effectiveness of therapeutic interventions.

## 5 Conclusion

CEUS significantly enhances the detection of vulnerable carotid plaques in patients with CRAO. Its integration with conventional ultrasound could improve embolic risk stratification and inform individualized secondary prevention strategies.

## Data Availability

The raw data supporting the conclusions of this article will be made available by the authors, without undue reservation.
